# Phosphorylation-Dependent Interactome of Ryanodine Receptor Type 2 in the Heart

**DOI:** 10.3390/proteomes9020027

**Published:** 2021-06-07

**Authors:** David Y. Chiang, Satadru Lahiri, Guoliang Wang, Jason Karch, Meng C. Wang, Sung Y. Jung, Albert J. R. Heck, Arjen Scholten, Xander H. T. Wehrens

**Affiliations:** 1 Cardiovascular Division, Department of Medicine, Harvard Medical School, Brigham and Women’s Hospital, Boston, MA 02115, USA; dychiang@bwh.harvard.edu; 2 Cardiovascular Research Institute, Department of Molecular Physiology & Biophysics, Baylor College of Medicine, Houston, TX 77030, USA; Satadru.Lahiri@bcm.edu (S.L.); glwang77@gmail.com (G.W.); Jason.Karch@bcm.edu (J.K.); 3 Department of Molecular Physiology & Biophysics, Baylor College of Medicine, Houston, TX 77030, USA; 4 Huffington Center on Aging, Department of Molecular and Human Genetics, Baylor College of Medicine, Houston, TX 77030, USA; wmeng@bcm.edu; 5 Department of Molecular and Human Genetics, Baylor College of Medicine, Houston, TX 77030, USA; 6 Howard Hughes Medical Institute, Baylor College of Medicine, Houston, TX 77030, USA; 7 Department of Biochemistry, Baylor College of Medicine, Houston, TX 77030, USA; syjung@bcm.edu; 8 Biomolecular Mass Spectrometry and Proteomics, Bijvoet Center for Biomolecular Research and Utrecht Institute for Pharmaceutical Sciences, Utrecht University, 3584 Utrecht, The Netherlands; a.j.r.heck@uu.nl (A.J.R.H.); ascholt1@its.jnj.com (A.S.); 9 Netherlands Proteomics Centre, 3584 Utrecht, The Netherlands; 10 Department of Medicine (Cardiology), Baylor College of Medicine, Houston, TX 77030, USA; 11 Department of Neuroscience, Baylor College of Medicine, Houston, TX 77030, USA; 12 Department of Pediatrics (Cardiology), Baylor College of Medicine, Houston, TX 77030, USA; 13 Center for Space Medicine, Baylor College of Medicine, Houston, TX 77030, USA

**Keywords:** RyR2, interactome, affinity-purification mass spectrometry, phosphorylation, atrial fibrillation, heart failure

## Abstract

Hyperphosphorylation of the calcium release channel/ryanodine receptor type 2 (RyR2) at serine 2814 (S2814) is associated with multiple cardiac diseases including atrial fibrillation and heart failure. Despite recent advances, the molecular mechanisms driving pathological changes associated with RyR2 S2814 phosphorylation are still not well understood. **Methods:** Using affinity-purification coupled to mass spectrometry (AP-MS), we investigated the RyR2 interactome in ventricles from wild-type (WT) mice and two S2814 knock-in mutants: the unphosphorylated alanine mutant (S2814A) and hyperphosphorylated mimic aspartic acid mutant (S2814D). Western blots were used for validation. **Results:** In WT mouse ventricular lysates, we identified 22 proteins which were enriched with RyR2 pull-down relative to both IgG control and no antibody (beads-only) pull-downs. Parallel AP-MS using WT, S2814A, and S2814D mouse ventricles identified 72 proteins, with 20 being high confidence RyR2 interactors. Of these, 14 had an increase in their binding to RyR2 S2814A but a decrease in their binding to RyR2 S2814D. We independently validated three protein hits, Idh3b, Aifm1, and Cpt1b, as RyR2 interactors by western blots and showed that Aifm1 and Idh3b had significantly decreased binding to RyR2 S2814D compared to WT and S2814A, consistent with MS findings. **Conclusion:** By applying state-of-the-art proteomic approaches, we discovered a number of novel RyR2 interactors in the mouse heart. In addition, we found and defined specific alterations in the RyR2 interactome that were dependent on the phosphorylation status of RyR2 at S2814. These findings yield mechanistic insights into RyR2 regulation which may guide future drug designs.

## 1. Introduction

Ryanodine receptor type 2 (RyR2) is the main intracellular calcium (Ca^2+^) channel which resides on the sarcoplasmic reticulum (SR) in cardiomyocytes, responsible for excitation–contraction (EC) coupling. In a process known as Ca^2+^-induced Ca^2+^ release, RyR2 senses Ca^2+^ influx from the L-type Ca^2+^ channel (LTCC) during cardiac systole and responds by opening to release a large amount of Ca^2+^ into the cytosol for contraction of the myofibrils. During diastole, RyR2 closes while intracellular Ca^2+^ is pumped back into the SR via sarcoplasmic/endoplasmic reticulum Ca^2+^-ATPase 2a (SERCA2a) and extruded from the cell via the Na^+^/Ca^2+^ exchanger (NCX). Because of its central role, RyR2 is tightly regulated by a myriad of regulating subunits, enzymes, and targeting/anchoring proteins in a macromolecular complex that exceeds three million daltons [1]. Conversely, dysregulation of RyR2 by a number of mechanisms can lead to inappropriate Ca^2+^ release during diastole (“RyR2 leak”), which is associated with several cardiac diseases including catecholaminergic polymorphic ventricular tachycardia, atrial fibrillation, and heart failure [2,3,4,5,6,7]. In particular, we and others have shown that hyperphosphorylation of RyR2 at serine 2814 (S2814) by Ca^2+^/calmodulin-dependent protein kinase II (CaMKII) contributes to these RyR2 leaks and is implicated in both atrial fibrillation and heart failure; by contrast, genetic ablation of S2814 (mutating to an alanine, S2814A) rescues disease phenotypes in mice [3,4,5]. Despite the elucidation of these and other pathogenic mechanisms involving RyR2, there is still a lack of viable therapeutic options targeting RyR2 function [2]. Therefore, there is significant interest in discovering and understanding novel regulators of this massive homotetrameric channel with the goal of developing novel therapeutic strategies [2].

Previously, we applied affinity-purification coupled to mass spectrometry (AP-MS) to study the RyR2 interactome in three different settings. In the first study, we immunoprecipitated RyR2 and junctophilin-2 (JPH2), a known regulator of RyR2, separately from mouse hearts and identified using MS a novel and common interactor of both proteins: striated muscle-specific serine/threonine protein kinase (SPEG) [8]. Cardiomyocyte-specific conditional knockout of *Speg* in mouse led to JPH2 dephosphorylation, disruption of transverse tubules, and development of heart failure [8]. Using the same mouse model, we performed a similar RyR2 AP-MS and identified serine 2367 on RyR2 as a novel kinase substrate of SPEG and showed in another mouse model that ablation of this phosphorylation site led to inappropriate RyR2 activity in atrial myocytes and increased susceptibility for atrial fibrillation [9]. In the third study, we immunoprecipitated RyR2 and identified a number of protein phosphatases using MS including a novel RyR2 interactor, protein phosphatase 1 (PP1)-regulatory subunit PPP1R3A, which when knocked out also led to inappropriate RyR2 activity and atrial fibrillation susceptibility in mouse [10]. Together, these studies established the viability and usefulness of such unbiased approaches in understanding macromolecular complexes such as RyR2.

In this study, we applied a similar AP-MS approach to probe the RyR2 interactome with respect to the phosphorylation status at S2814, given its importance in the pathogenesis of multiple cardiac diseases. To do this, we took advantage of two mouse models our lab had previously generated: the RyR2 S2814A mouse [7] (ablation of the S2814 phosphorylation site) and the RyR2 S2814D mouse [6] (where serine is mutated to aspartic acid to mimic constitutive phosphorylation of RyR2 at 2814). By comparing the RyR2 interactome in ventricular lysates from wild-type (WT), S2814A, and S2814D mice, we were able to not only discover novel RyR2 interactors but particularly interactors with differential binding to RyR2 based on the phosphorylation status at S2814. We believe that these findings reveal new insights into the regulation of RyR2 and may guide future mechanistic studies and drug designs.

## 2. Materials and Methods

### 2.1. Study Animals

All animal studies were performed according to protocols approved by the Institutional Animal Care and Use Committee of Baylor College of Medicine conforming to the *Guide for the Care and Use of Laboratory Animals* published by the U.S. National Institutes of Health. RyR2 S2814A [7] and S2814D [6] knock-in mice were previously generated and were backcrossed onto the C57Bl/6J background for >10 generations.

### 2.2. Lysate Preparation

Whole mouse ventricles were homogenized in modified radioimmunoprecipitation assay (RIPA) buffer containing 150 mM NaCl, 10 mM Tris-HCl, 20 mM NaF, 1 mM NaVO_3_, protease and phosphatase inhibitor cocktails (cOmplete, Mini and PhosSTOP, Roche Applied Science, Penzberg, Germany), and 1% CHAPS (3-[(3-cholamidopropyl) dimethylammonio]-1-propanesulfonate), with pH adjusted to 7.4. Samples were further sonicated (3 times for 1 s each) and centrifuged at 16,000 RPM at 4 °C for 15 min, and the supernatants were collected as lysate and used for downstream immunoprecipitation.

### 2.3. Co-Immunoprecipitation, Gel Electrophoresis, and Protein Digestion

These steps were carried out using protocols similar to those previously described [11]. In brief, ventricular lysates were precleared with Protein G Plus Agarose beads (Thermo Fisher Scientific, Waltham, MA, USA) for 2 h at 4 °C and separated into 3 equal fractions for incubation overnight at 4 °C with either anti-RyR2 mouse antibody (MA3-916; Thermo Fisher), IgG isotype control antibody (M5284; Sigma-Aldrich, St. Louis, MO, USA), or no antibody (beads-only control). The next morning, the samples were incubated with Protein G Plus Agarose beads for 1 h at 4 °C on a rotator, and the beads were subsequently washed 3 times with RIPA buffer without CHAPS. The beads were then incubated in Laemmli sample buffer with β-mercaptoethanol either at 70 °C for 10 min or room temperature for 30 min before the samples were run on a gradient SDS-PAGE gel. The gels were fixed and stained with bio-safe Coomassie dye (Bio-Rad Laboratories, Hercules, CA, USA), and each sample lane on the gels was cut into ten equally spaced pieces, which were subsequently reduced, alkylated, and in-gel digested as previously described [11].

### 2.4. Mass Spectrometry, Protein Identification, and Quantitation

Nanoscale liquid chromatography coupled to tandem mass spectrometry (LC-MS/MS) was performed as described [11]. Protein identification and quantitation were also performed as described [11]. In brief, the MS output files were loaded into MaxQuant and Proteome Discoverer and searched with mouse FASTA file from UniProt. Proteins that were only identified by site, reverse hits, or contaminants were filtered out. In addition, all immunoglobulin-related and keratin-related hits were removed. For MaxQuant analysis, only proteins with 3 or more unique peptides were kept. Label-free quantification (LFQ) intensities were used for comparison between samples that were ran in parallel [12]. For proteins with an intensity value of 0, an arbitrary minimum (background) intensity of 50,000 was used for ratio calculations. For Proteome Discoverer analysis, PSM (peptide spectrum matches) were quantitated; hits are required to be found in at least one of the WT, S2814A, and S2814D samples with PSM ≥ 5 and with at least 2 peptides identified. The PSM of each hit from all samples is normalized to the WT RyR2 PSM. 

### 2.5. Co-Immunoprecipitation and Western Blotting

Ventricular lysates were pre-cleared with Pierce Protein A/G Plus Agarose beads (Thermo Fisher Scientific, Waltham, MA, USA) for 2 h at 4 °C and separated into 2 equal fractions for incubation overnight at 4 °C with either anti-RyR2 mouse antibody (custom antibody, Rabbit, YenZym, Brisbane, CA, USA) or rabbit IgG isotype control antibody (12-370; Sigma-Aldrich, St. Louis, MO, USA). The next morning, samples were incubated with Pierce Protein A/G Plus Agarose beads for 2 h at 4 °C on a rotator, and the beads were subsequently washed 2 times with RIPA buffer (first wash without CHAPS and second wash with 0.5% CHAPS). The beads were then incubated in 2X Laemmli sample buffer (1610737, Biorad, Hercules, CA, USA) with 5% β-mercaptoethanol either at 70 °C for 10 min or room temperature for 30 min before the samples were run on a gradient SDS-PAGE gel (stacking–4%, resolving–5% and 8%). Following gel running, transfer to PVDF membrane was done overnight (~14 hrs) at constant 20 volts. Membranes were blocked with OneBlock Western-CL blocking buffer (20-313, Genesee Scientific, San Diego, CA, USA) for 1 h at room temp. Primary antibody incubation was done overnight at 4 °C, followed by 3 TBST (1% Tween-20) washes, secondary antibody incubation for 1 h at room temp, and membrane developing with LiCor Odyssey imaging system. Protein bands of interest were quantified using the ImageJ software. Primary antibodies used were against RyR2 (rabbit polyclonal, custom made against mouse RyR2, YenZym, 1:1000 for IP); RyR2 (MA3-916, mouse monoclonal, Thermo Fisher Scientific, 1:4000 for IB); Cpt1B (SC-393070, mouse monoclonal, Santa Cruz Biotechnologies, Santa Cruz, CA, USA; 1:1000 for WB); Aifm1 (SC-13116, mouse monoclonal, Santa Cruz, 1:1000 for IB) and Idh3b (A305-501A-M, rabbit polyclonal, Thermo Fisher Scientific, 1:1000 for IB). Secondary antibodies used were anti-mouse Alexa-Fluor-680 (A-2105, goat polyclonal, Thermo Fisher Scientific, 1:15,000), anti-rabbit Alexa-Fluor-800 (A-32735, goat polyclonal, Thermo Fisher Scientific, 1:15,000).

### 2.6. Western Blot Analysis

Western blot images were analyzed using ImageJ. Bands were labeled with same size of box (using rectangle tool) and area under the curve from the histogram was quantified. Co-immunoprecipitated proteins were normalized to immunoprecipitated RyR2 (interacting protein/RyR2 IP). These values from WT, S2814A, and S2814D mice were further normalized to WT before plotting in Prism (GraphPad Software, San Diego, CA, USA).

### 2.7. Statistics

Results are expressed as mean ± standard error of the mean (SEM). One-way ANOVA test was used for comparisons between WT, S2814A, and S2814D groups followed by multiple comparison. A *p* value of less than 0.05 was considered statistically significant.

## 3. Results

### 3.1. Identification of Novel RyR2 Interactors

**Figure 1:**
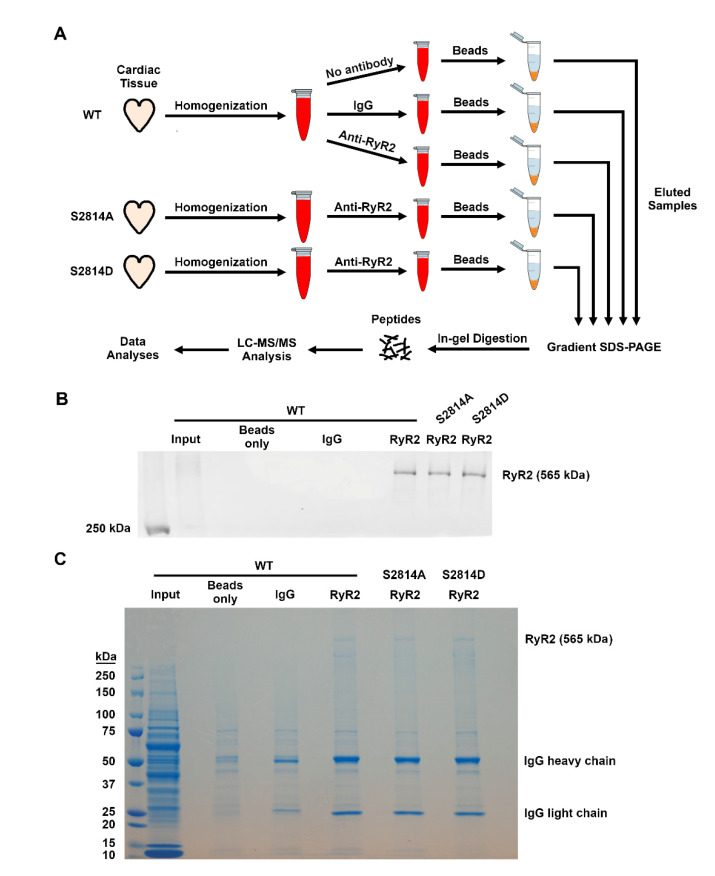
RyR2 affinity-purification coupled to mass spectrometry. (**A**). Schematic showing the major methodological steps. LC = liquid chromatography; MS/MS = tandem mass spectrometry (**B**). Western blot validation of the immunoprecipitation of RyR2 from WT, RyR2 S2814A, and S2814D mouse ventricles. (**C**). Coomassie staining of gel containing samples from the various immunoprecipitations. Each lane (except for the input lane) is cut into ten equal pieces for in-gel digestion and LC-MS/MS analysis in parallel.

To evaluate the RyR2 interactome in an unbiased fashion, we applied a similar AP-MS method as previously described with modifications (Figure 1A). Using whole ventricles harvested from WT, S2814A, and S2814D mice, we immunoprecipitated RyR2 across these samples in parallel and validated the efficiency of the pull-down by western blotting (Figure 1B). Once the RyR2 IP was confirmed by western blots, we ran the remaining samples on a full gel to achieve complete protein separation and stained the gel with Coomassie blue for visual confirmation (Figure 1C). Each lane of the gel (except input) was subsequently cut into ten pieces for in-gel digestion and downstream LC-MS/MS analysis (Figure 1A).

**Figure 2:**
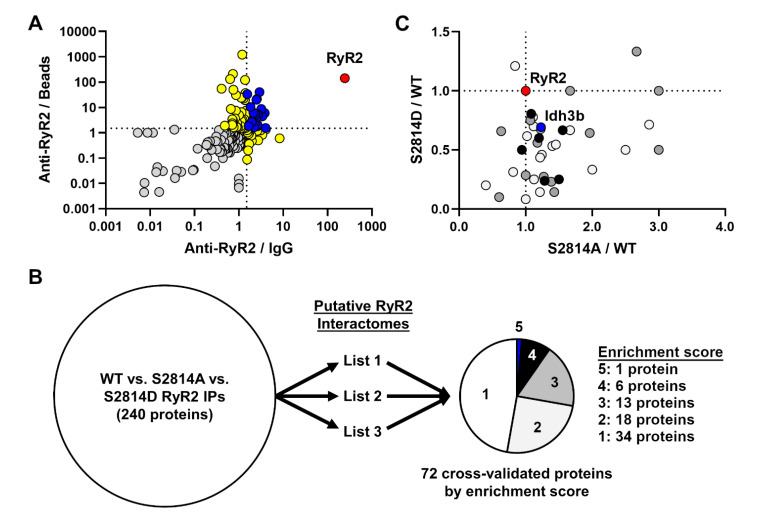
Phosphorylation-dependent alterations in cardiac RyR2 interactome. (**A**) RyR2 interactome defined based on enrichment with RyR2 pull-down compared to IgG (anti-RyR2/IgG) and beads-only (anti-RyR2/Beads) pull-downs. Ratios for each identified protein were calculated based on label-free quantification (LFQ) intensities across samples which were run and analyzed in parallel. Dotted lines mark ratios of 1.5, above which there is considered enrichment with RyR2 pull-down. The bait protein RyR2 (red) is highly enriched in the pull-down. Blue marks proteins enriched in RyR2 pull-down with respect to both IgG and beads-only controls (22 proteins). Yellow marks proteins enriched in RyR2 pull-down with respect to either IgG (15 proteins) or beads-only control (42 proteins). (**B**) Cross-validation and prioritization of WT, S2814A, and S2814D RyR2 IPs using published (List 1) [8] and unpublished (Lists 2 [10] and 3) putative RyR2 interactomes. List 3 is the putative RyR2 interactome generated in this study (Table S1). Enrichment score calculation is described in the Results section. (**C**). Alterations in the RyR2 interactome based on the phosphorylation-mimic status at S2814. Ratios for each protein were calculated based on peptide spectrum matches (PSM) and plotted for S2814A mutant over WT (S2814A/WT) and S2814D mutant over WT (S2814D/WT). Dotted lines mark ratios of 1.0. Each protein was cross-validated against three RyR2 AP-MS experiments to generate an enrichment score (the higher the score, the more likely it is a true RyR2 interactor) with the maximum score being 6 (see panel B). White, grey, and black circles are proteins with a score of 2, 3, and 4, respectively. The blue circle marks the only protein with a score of 5 (Idh3b), whereas the red circle marks the bait protein RyR2.

To identify putative RyR2 interactors, we first compared the 3 WT samples subjected to anti-RyR2 antibody, IgG control antibody, or no antibody control (beads only). We searched the MS hits from these samples using MaxQuant which identified 181 proteins after filtering (Table S1). For each protein, we obtained their LFQ intensities for each of the three samples and took the ratios between anti-RyR2 versus IgG control (RyR2/IgG) and anti-RyR2 versus beads-only control (RyR2/Beads). Figure 2A shows the plot of these ratios for each protein, demonstrating strong enrichment of the bait protein RyR2 (red symbol) and 22 proteins where the RyR2/IgG and RyR2/Beads ratios were both greater than 1.5 (blue symbols). Of the remaining proteins, 15 were enriched with RyR2 pull-down with respect to IgG control, and 42 were enriched with respect to beads-only control (both yellow symbols). These data are summarized in Table S1.

### 3.2. Effect of RyR2 S2814 Phosphorylation Status on Protein–Protein Interactions

After establishing the putative RyR2 interactome, we next asked how this interactome is altered based on the phosphorylation status at RyR2 S2814. To do this, we compared the PSM counts for each protein between RyR2 IP samples from WT, S2814A, and S2814D mouse hearts. After quality control, we identified a total of 240 proteins with PSM counts in at least one of the three samples. However, because this list contained a mix of specific and nonspecific interactors from the pull-down, it required further filtering and cross-validation against established RyR2 interactome(s) such as the one generated above (Table S1). Because RyR2 is such a large protein and a membrane protein in an intracellular organelle, the SR, co-IP of RyR2 is particularly challenging and difficult to replicate unless experimental conditions are nearly identical. For this reason, we decided to take advantage of two similar RyR2 AP-MS experiments we conducted in the past (one published [8] and one unpublished [10]) in order to increase the stringency of the cross-validation (Figure 2B). Altogether, we have three lists for cross-validation: the published list from Quick et al. [8] (List 1) the unpublished list from Alsina *et al.* [10] (List 2), and the newly generated list from this study (List 3; Table S1).

From each of the 3 RyR2 AP-MS experiments, we have anti-RyR2/IgG and anti-RyR2/beads LFQ intensity ratios, for a total of 6 such “enrichment ratios” (two from each AP-MS experiment). Using 1.5 as the cut-off, each protein is given an “enrichment score” based on the number of enrichment ratios it has above 1.5, for a maximum score of 6 (since there are only 6 such ratios). In this way, we could rank our confidence on each identified protein based on the number of times it had been enriched in these three RyR2 AP-MS experiments. Using this strategy, we were able to score 72 proteins besides the bait protein RyR2 (Figure 2B; Table S2). Thirty-four and 18 proteins have a score of 1 and 2, respectively and are considered low confidence RyR2 interactors. Thirteen and six proteins have a score of 3 and 4, respectively, and are considered high confidence RyR2 interactors. Only one protein, Idh3b, had a score of 5, besides the bait RyR2 itself (Figure 2B). Together, this yielded 20 high confidence putative RyR2 interactors.

Once we cross-validated and ranked the list of putative interactors, we examined the level of their binding to RyR2 with respect to the phosphorylation-mimic status at S2814. To do this, we compared the normalized PSM count of each protein between the WT versus S2814A (S2814/WT) and WT versus S2814D (S2814D/WT) samples and plotted these ratios for proteins with an enrichment score of 2 or higher (Figure 2C). The majority of RyR2 interactors are located in the lower-right quadrant in Figure 2C, suggesting that they have an increased binding to RyR2 S2814A mutant (S2814A/WT > 1.0) but decreased binding to RyR2 S2814D mutant (S2814D/WT < 1.0). In other words, phosphorylation-mimic at S2814 decreases binding of the majority of putative RyR2 interactors found here, whereas the ablation of the phosphorylation site at S2814 had the opposite effect. Interestingly, a minority of proteins moved in the same direction (either both up or down) for both S2814A and S2814D mutants (Figure 2C). These findings suggest that the phosphorylation state at S2814 plays a crucial role in determining the RyR2 interactome and that hyperphosphorylation of S2814 may lead to significant alterations in this interactome.

### 3.3. Validation of Select RyR2 Interactors

To corroborate our AP-MS findings, we selected three proteins for validation with co-IP followed by western blots, based on the prioritized rank list (Table S2) and relevance to muscle physiology. As shown in Figure 2B,C, Idh3b (isocitrate dehydrogenase 3, beta subunit) is the only interactor with an enrichment score of 5. It is associated with an autosomal-recessive form of retinitis pigmentosa [13]. Aifm1 (apoptosis-inducing factor, mitochondrion-associated 1) has an enrichment score of 4 and relatively high PSM counts. It is associated with a number of X-linked recessive disorders including encephalomyopathy [14,15]. Cpt1b (carnitine O-palmitoyltransferase 1, muscle isoform) has an enrichment score of 3 with high PSM counts and its deficiency in mouse exacerbates pressure overload-induced cardiac hypertrophy and heart failure [16]. All three putative interactors had increased binding to RyR2-S2814A and decreased binding to RyR2-S2814D compared to WT (Table S2).

**Figure 3:**
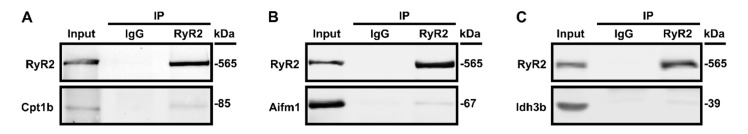
Validation of RyR2 interactors identified from mass spectrometry. Western blot of RyR2 immunoprecipitated (IP) lysates from wild-type (WT) mouse ventricles showing RyR2 co-immunoprecipitation (co-IP) with Cpt1b (**A**), Aifm1 (**B**), and Idh3b (**C**). Mouse IgG was used as a negative control for these co-IP experiments. Representative blots from 3 independent experiments.

**Figure 4:**
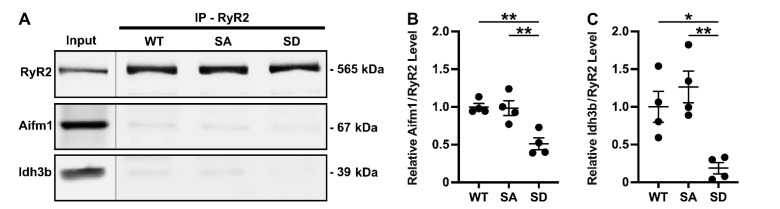
RyR2 S2814 phosphorylation-dependent interaction with Aifm1 and Idh3b. Western blot of RyR2 immunoprecipitated (IP) from lysates of ventricles from wild-type (WT), S2814A (SA), and S2814D mice showing differential RyR2 co-immunoprecipitation (co-IP) with Aifm1 and Idh3b (**A**). Quantification of co-immunoprecipitated proteins normalized to immunoprecipitated RyR2 (relative to WT) showing significantly reduced RyR2/Aifm1 (**B**) and RyR2/Idh3b (**C**) interactions in S2814D mice compared to both WT and S2814A mice. * *p* < 0.05; ** *p* < 0.01.

**Figure 5:**
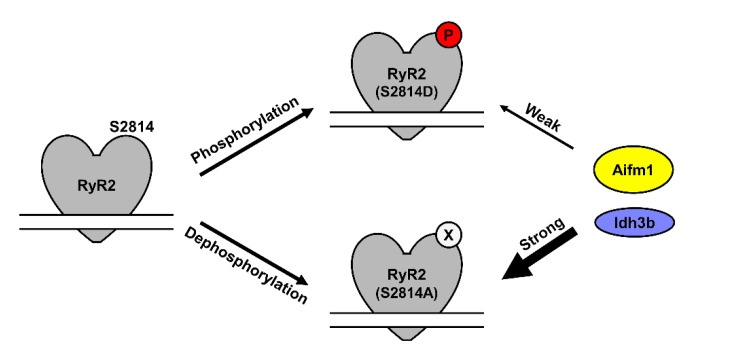
Changes in the RyR2 interactome based on phosphorylation status of S2814. As representatives of other putative interactors, Aifm1 and Idh3b have decreased binding to RyR2 S2814D mimicking phosphorylated RyR2 at S2814 (“Weak” arrow) but increased or “Strong” binding to RyR2 S2814A mimicking dephosphorylated RyR2 at S2814.

Figure 3 shows western blots of these three proteins following RyR2 IP from WT mouse ventricular lysates, demonstrating specific co-IP of all three proteins with RyR2. After confirming their interaction with RyR2, we repeated the RyR2 IPs using WT versus S2814A and S2814D samples. As shown in Figure 4, the RyR2 IP efficiency is similar across these three samples, and the relative bindings of Aifm1 and Idh3b recapitulated the AP-MS findings. Although there was no statistical difference in their binding between the WT and S2814A samples, both proteins were significantly decreased in their binding to RyR2 S2814D, relative to both WT and S2814A RyR2. This trend is consistent with the AP-MS findings as depicted in Figure 2C. For Cpt1b, the IPs across WT, S2814A, and S2814D were inconclusive, requiring further optimization, although the same trend was observed (data not shown). These experiments validated our AP-MS findings which showed that RyR2 interactome is altered based on S2814 phosphorylation status (Figure 5).

## 4. Discussion

In this study, we applied a state-of-the-art proteomic approach to understand the RyR2 interactome in the heart and discovered a number of novel interactors. In addition, we found and defined specific alterations in the RyR2 interactome that were dependent on the phosphorylation status of RyR2 at one single CaMKII phosphorylation site, S2814, which has previously been implicated in several cardiac diseases. Altogether, our findings shed new mechanistic insights into the regulation of RyR2 and open new avenues for future research, as detailed below.

### 4.1. Quantitative Affinity Purification Mass Spectrometry

Protein–protein interactions are central to biological function and cellular processes in all tissues and cell types. In order to study these interactions at scale and in an unbiased way, several technologies have been developed in recent decades including protein microarrays, phage display, and the yeast two-hybrid systems. However, all of these approaches rely on *in vitro* assays or heterologous biological systems, which limit the relevance of their findings [17]. By contrast, affinity purification combined with mass spectrometry allows for unbiased detection of protein–protein interactions under both physiological conditions and relevant biological contexts including cell lines and even primary tissues, as we have previously shown using human atrial biopsies [11] and mouse ventricles [18]. Moreover, with advances in analytical methods, AP-MS experiments can provide quantitative information which greatly increases the confidence of interactor identification and allows for study of interactions under experimental perturbations, as we have demonstrated in this manuscript. Therefore, quantitative AP-MS is arguably one of the most powerful technologies to identify and study protein–protein interactions in health and disease [17].

### 4.2. Phosphorylation-Dependent Protein–Protein Interactions

An important subset of protein–protein interactions depends on the phosphorylation state of the target protein. In many cases, phosphorylation changes the conformation, activity, localization, and/or stability of the target protein, all of which may affect its interactions with other proteins. One well-studied mechanism is when the phosphorylated residue creates binding sites for other phosphoprotein-binding domain, leading to increased protein–protein interaction. For example, the Src-homology-2 (SH2) domains found in many tyrosine kinases bind exclusively to phospho-tyrosine, whereas 14-3-3, polo-like kinase 1 (Plk1), and Skp2 bind to serine/threonine phosphorylated residues [19]. Although many studies have applied mass spectrometry to studying protein phosphorylation (i.e., phospho-proteomics) [20], very few groups have quantitated phosphorylation-dependent interactions unbiasedly at scale. In one study, the authors performed a co-IP with phosphorylated tau from neurofibrillary tangles of patients with Alzheimer’s disease and identified 75 proteins that interacted with phosphorylated tau [21]. However, the negative control used was nonspecific IgG instead of unphosphorylated tau; therefore, the interactors identified may not truly be phosphorylation-dependent.

In our study, we took advantage of two RyR2 knock-in mutants at S2814 which mimic unphosphorylated (S2814A) [7] and constitutively hyperphosphorylated (S2814D) [6] RyR2; the latter of which mimics high S2814 phosphorylation seen in multiple cardiac diseases including atrial fibrillation and heart failure [3,4,5]. Instead of finding an increase in interactors with S2814D, we found that the majority of interactors had a decreased interaction with RyR2, when compared to WT and S2814A mutant (Figure 2B; Table S2). This suggests that the underlying mechanism may be different from the ones discussed above where interactors recognize and bind phosphorylated residues [19]. In this case, it is conceivable that S2814 phosphorylation leads to conformation changes that affect protein–protein interactions elsewhere in the protein. In any case, the majority of the changes found moved in opposite directions between the S2814A and S2814D mutants, with S2814A behaving more similarly to WT, which are consistent with physiological data from these mouse models [3,4,5]. Although we did not quantify baseline phosphorylation levels of RyR2 in WT mice, prior studies suggest that baseline levels of RyR2 phosphorylation at S2814 are low [5,22]. To the best of our knowledge, this is the first AP-MS study that employed genetic perturbations to studying phosphorylation-dependent interactome changes.

### 4.3. CaMKII Phosphorylation-Dependent RyR2 Interactions

Previous studies have established that CaMKII-mediated phosphorylation at RyR2 S2814 leads to channel opening and SR Ca^2+^ leak. Single-channel studies further revealed an increased open probability of the phosphomimetic RyR2 S2814D mutant channel compared to WT or nonphosphorylated S2814A mutant [6]. At the resting closed state, RyR2 N-terminal (0–600 aa) and central (2000–2500 aa) regions interact with each other in a conformation known as zipping [23]. This interaction is critical to maintaining RyR2 stability as disease-related mutations at these domains unzip RyR2 leading to an unstable open (leaky) state predominantly observed during heart failure. However, the underlying mechanism of this modification is not well understood [24,25]. A synthetic peptide DPc10 (2460–2495 aa) can destabilize RyR2 acting as a competitive binding inhibitor at the RyR2 N-terminal region, further disrupting the zipping of RyR2 [26]. Using DPc10 in mouse myocytes, one study showed that acute CaMKII activation of wild-type RyR2 or phosphomimetic RyR2 S2814D can both induce a pathological conformational change in RyR2 (detected via increased access of the RyR2 structural peptide DPc10), associated with increased SR Ca^2+^ leak [27]. This study further showed that increased RyR2 S2814 phosphorylation is associated with the reduced affinity between RyR2 and stabilizing partner calmodulin (CaM). Similarly, we found that the majority of putative RyR2 interactors had decreased binding to the phosphomimetic S2814D, possibly due to its conformational change. Future mechanistic studies are needed to determine if these novel RyR2 interactors are critical to the closed-state stability and function of RyR2.

### 4.4. Novel Phosphorylation-Dependent RyR2 Interactors

Our studies revealed several previously unknown RyR2 binding proteins, including Aifm1, Cpt1b, and Idh3b. These top scoring proteins are all mitochondrial proteins, which may not be completely surprising. Calcium plays a major role in regulating endoplasmic reticulum and mitochondrial function, and structural and functional connections between these organelles have been reported in some muscle cell types [28]. In addition, augmented RyR2 activity—such as the one caused by excessive S2814 phosphorylation—can modulate mitochondrial Ca^2+^ handling, promoting reactive oxygen species emissions and cell death, and exacerbating the development of cardiac disease [29,30,31,32].

Apoptosis-inducing factor mitochondrion-associated 1 (Aifm1 or AIF) is one of the most highly predicted and validated RyR2 interactors found in our study. Aifm1, a flavoprotein primarily located in mitochondrial intermembrane space (IMS), has a paradoxical dual role in mediating oxidative phosphorylation with its oxidoreductase activity and in inducing caspase-independent DNA degradation when it migrates to the nucleus following proapoptotic stimuli [33,34,35]. In healthy cells, most of the cytosolic Aifm1 proteins get cleaved at the N-terminus to translocate into the IMS to regulate mitochondrial respiration and energy homeostasis [36,37]. During a stress that induces loss of mitochondrial membrane potential, calpain-1 mediated cleavage releases mitochondrial Aifm1 which further translocates into the nucleus to induce chromatin condensation and DNA degradation in a process known as ‘parthanatos’ [38,39,40]. Loss of Aifm1 leads to a severe defect in mitochondrial respiration, primarily in complex I activity [13,41]. On the other hand, overexpression of Aifm1 leads to increased cell death in hypoxia-neuronal injury [42].

Primary cytosolic interactors of Aifm1 to date include heat-shock 70-kDa protein (HSP70), cyclophilin A (CypA), macrophage migration inhibitory factor (MIF), thioredoxin (Trx1), and eukaryotic translation initiation factor 3 subunit G (eIF3g) [33]. A couple of cardiac studies demonstrated that loss of Aifm1 leads to severe dilated cardiomyopathy in mice, mitochondrial dysfunction, and increased oxidative stress in cardiomyocytes [43,44]. Interestingly, we found this novel RyR2/Aifm1 interaction to be RyR2 S2814 phosphorylation-dependent, as increased S2814 phosphorylation ablates this interaction in S2814D mice. CaMKII is the primary kinase which phosphorylates RyR2 S2814 [5,22]. RyR2 S2814D mice with hyperphosphorylation at this site develop SR Ca^2+^ leak, delayed after depolarization, arrhythmogenesis leading to atrial fibrillation, whereas S2814A mice (phosphorylation-ablated) were protected. The S2814D mice were also prone to cardiomyocyte cell death following ischemia injury compared to WT and S2814A mice [30]. Previous studies further established a critical role of CaMKII in mediating cardiac cell death, although a direct involvement of Aifm1 downstream of CaMKII has yet to be established in the heart [45]. A novel RIP3-CaMKII-Aifm1 axis in regulating necroptosis was shown in one neurodegenerative disorder study [46]. Based on prior studies on the pro-parthanatos function of Aifm1 in the nucleus, it is critical to prevent Aifm1 translocation to the nucleus to inhibit necrosis. We hypothesize that RyR2 sequesters Aifm1 in the SR through this novel interaction, further preventing its nuclear translocation. Hyperphosphorylation at RyR2 S2814 in S2814D mice may reduce this interaction releasing Aifm1 to migrate to the nucleus and induce parthanatos. In addition, increased cytosolic Ca^2+^ in S2814D mice due to SR Ca^2+^ leak could potentially activate calpain-1 to further promote mitochondria to nucleus transition of proapoptotic form of Aifm1 and may also contribute to the loss of mitochondrial membrane potential through the opening of the mitochondrial permeability transition pore [40]. However, in-depth mechanistic studies are required to establish these novel concepts.

Carnitine palmitoyltransferase 1b (Cpt1b) emerged as another novel RyR2 interactor in this study. Cpt1b is the muscle isoform of the carnitine palmitoyltransferase protein family predominantly expressed in cardiac and skeletal muscle [47]. Cpt1b acts as a rate-limiting enzyme for mitochondrial fatty acid oxidation in the heart [48,49]. This mitochondrial outer-membrane protein mediates mitochondrial import of long-chain fatty acids as the first step in fatty acid oxidation, an essential process for cardiac metabolism [49]. Cpt1b mediates the esterification of long-chain fatty acyl-CoA to long-chain acylcarnites (LCACs), which further migrate from cytosol to the inner mitochondrial membrane [49]. As cardiac metabolism shifts from fatty acid oxidation to glycolysis during heart failure, Cpt1b-targeted therapy was used as a therapeutic approach in inhibiting fatty acid oxidation to treat heart failure [50]. However, preclinical studies with Cpt1b inhibitors were inconclusive [51,52]. Further mechanistic studies revealed that inhibition of Cpt1b actually promotes cardiac hypertrophy and heart failure [16,53], which may explain the inconclusive results of these preclinical studies. Our MS data showed that RyR2/Cpt1b interaction might be RyR2 S2814 phosphorylation-dependent, although the western blot validation was inconclusive. Several studies have shown that overload of LCACs promotes oxidative stress, SR Ca^2+^ leak, arrhythmia, and heart failure [54]. In particular, one study showed that LCACs oxidize RyR2, resulting in increased SR Ca^2+^ leak [55], thereby providing a direct link to ventricular arrhythmias, although the underlying mechanism has not been elucidated. An in-depth assessment of the RyR2/Cpt1b interaction and its downstream effect could potentially address these questions.

Finally, isocitrate dehydrogenase 3, beta subunit (Idh3b), is another novel RyR2 interactor identified in this study. Idh3b is an essential NAD^+^ dependent enzyme of the tricarboxylic acid (TCA) cycle, which catalyzes the oxidative decarboxylation of isocitrate to 2-oxoglutarate [56,57]. Homozygous loss-of-function mutation of Idh3b is associated with mitochondrial dysfunction in retinitis pigmentosa [13]. Interestingly, Idh3b was found to be downregulated at the mRNA level in a canine model of atrial fibrillation [58] and also dysregulated in left atrial tissue from patients with atrial fibrillation [59]. However, the role of this protein in the heart has not been studied to date. Therefore, future studies are needed to investigate the role of the RyR2/Idh3b interaction in cardiac physiology and pathophysiology, especially with respect to atrial fibrillation.

### 4.5. Clinical Implications

To our knowledge this study is the first investigation of the phosphorylation-dependent RyR2 interactome. We found that CaMKII-mediated phosphorylation of RyR2 at S2814 site reduces RyR2 interaction with Aifm1 and Idh3b, suggesting the vital role of these novel binding partners in maintaining Ca^2+^ signaling. Future mechanistic studies are needed to elucidate their roles in cardiomyocytes and determine if these interactions are critical for RyR2 function. Established RyR2 interacting partners FKBP12.6 and CaM maintain the stable closed state of the RyR2 channel during diastole, which is a therapeutic strategy for arrhythmias by blocking pathological SR Ca^2+^ leaks. Several preclinical studies have utilized these RyR2-binding proteins to stabilize RyR2 in treating arrhythmia and heart failure [60,61]. It is conceivable that the novel RyR2 interactors identified in this study may be similarly exploited in clinical settings, although thorough mechanistic studies are warranted. For example, one may hypothesize that increased interactions with these novel partners inhibit SR Ca^2+^ leak by stabilizing RyR2 closed state. If true, then AAV-mediated delivery of genes or small molecules that enhance these interactions may become novel therapeutic strategies for the treatment of heart failure and cardiac arrhythmias.

## 5. Conclusions

In this study, we applied state-of-the-art proteomic methods to studying the RyR2 interactome in the heart. In addition to discovering a number of novel RyR2 interactors, we identified a number of specific alterations in the RyR2 interactome that were dependent on the phosphorylation status of RyR2 at S2814. These findings shed novel mechanistic insights into RyR2 regulation and open new avenues for future research and therapeutic considerations, specifically how RyR2 phosphorylation might regulate SR-mitochondrial signaling.

### Potential Limitations

Phosphomimetic residues do not completely recapitulate phosphorylation events both in terms of size and charge. Therefore, if the phosphorylation site serves as a recognition signal for an adaptor protein such as 14-3-3, the mutant may not bind to the adapter protein [20]. However, the fact that we found a general decrease in interactor bindings with the phosphomimetic S2814D suggests that the mechanism of alteration may be at sites other than S2814. In addition, because these mutant mice are germline genetic mutants, they may have other global and chronic proteomic changes that could have affected the RyR2 interactome independent of the phosphorylation-mimic status at S2814. As best as we could tell, however, their cardiac physiology is unchanged at baseline without stress [6].

## Data Availability

The MS data have been deposited to the ProteomeXchange Consortium via the PRIDE partner repository with the dataset identifier PXD026350.

## Supplementary Materials

The following are available online at https://www.mdpi.com/article/10.3390/proteomes9020027/s1, Table S1: Putative RyR2 interactors identified by AP-MS, Table S2: Phosphorylation-dependent changes in RyR2 interactome. File S1: Original Images for Blots/Gels.
