# Supplementary figures and images for: Phosphorylation-Dependent Interactome of Ryanodine Receptor Type 2 in the Heart
Source: Proteomes. 2021 Jun 7;9(2):27. doi: 10.3390/proteomes9020027 (PMC8293434; doi:10.3390/proteomes9020027)

**Fig 1B**

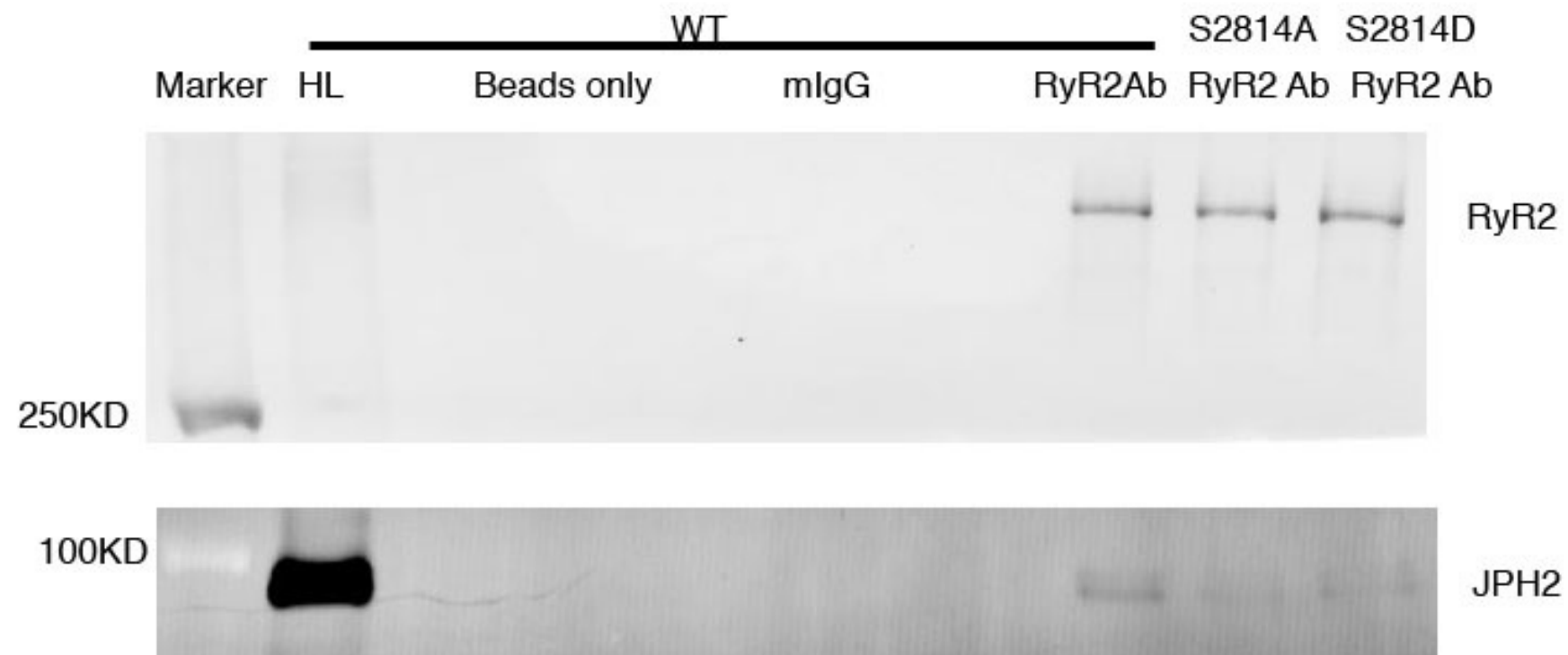

# Fig 1C

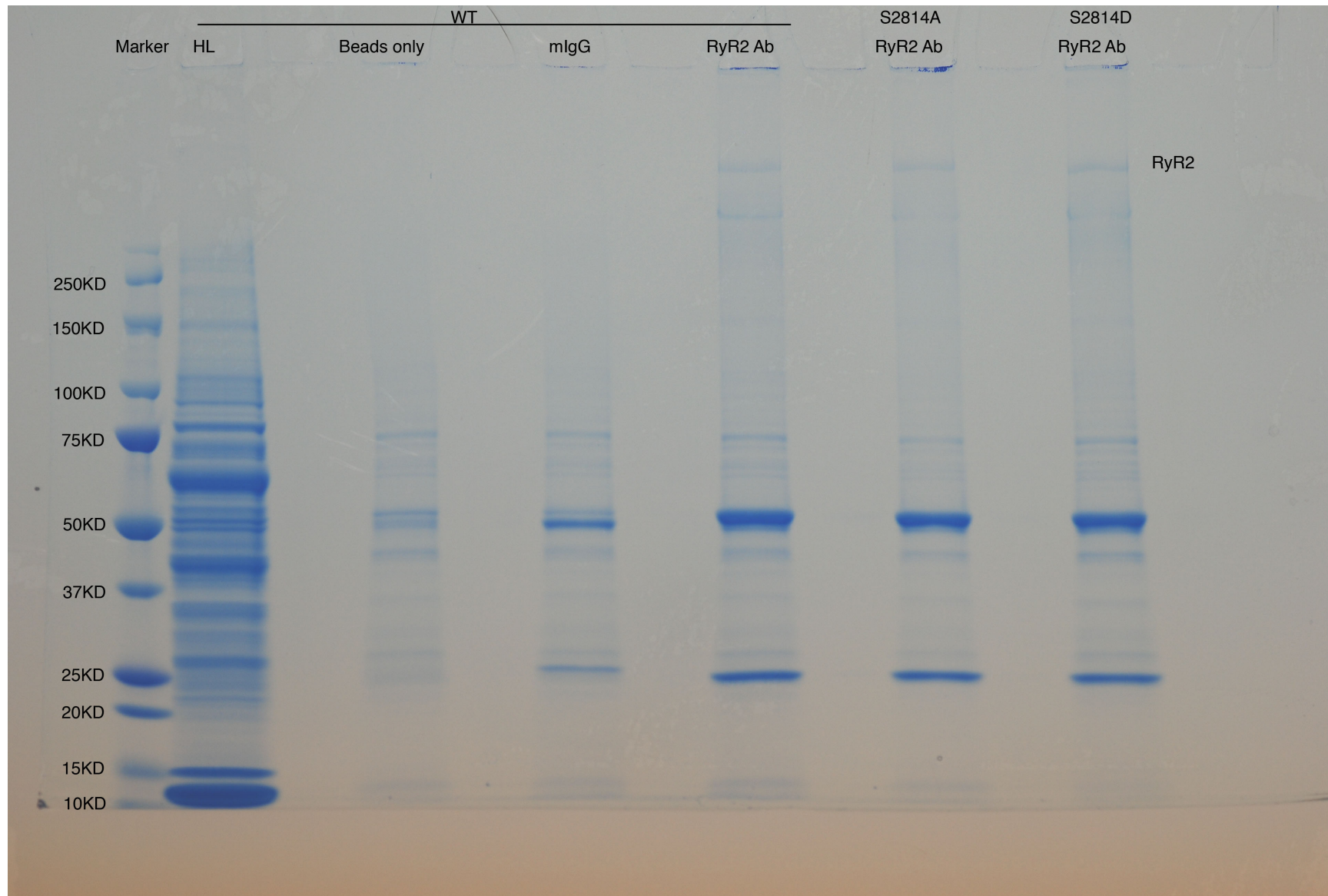

# Fig 3A

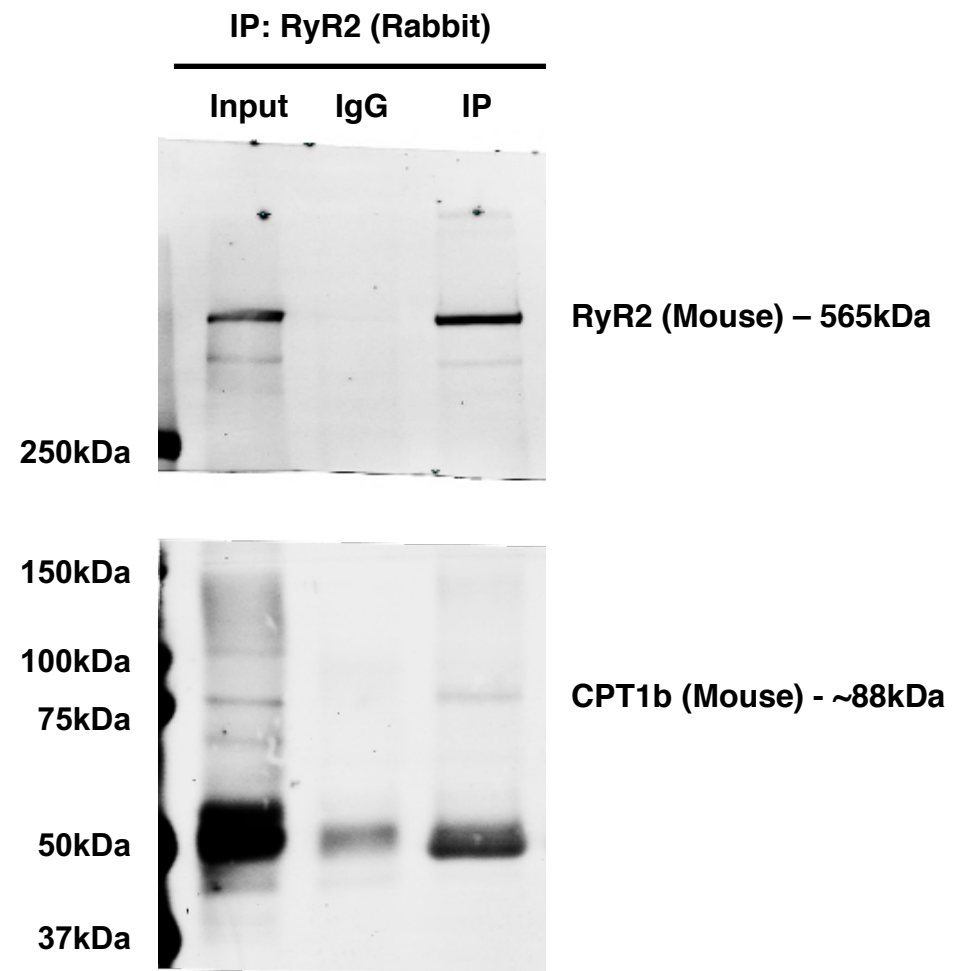

# Fig 3B

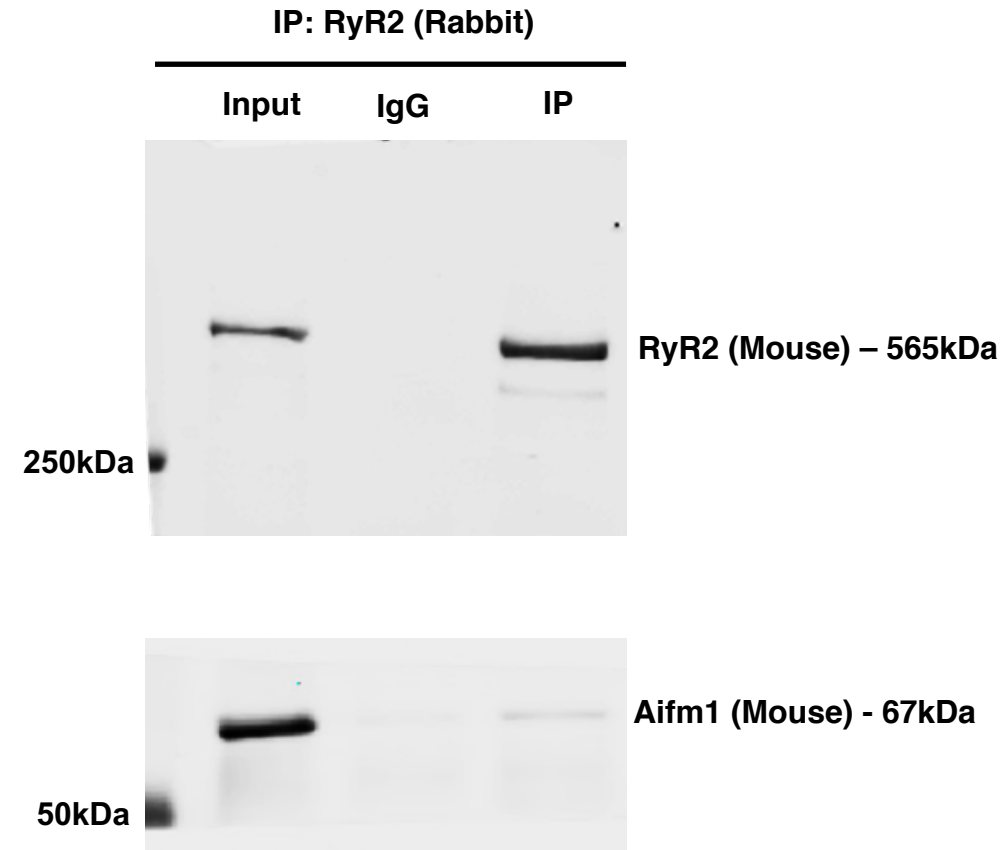

# Fig 3C

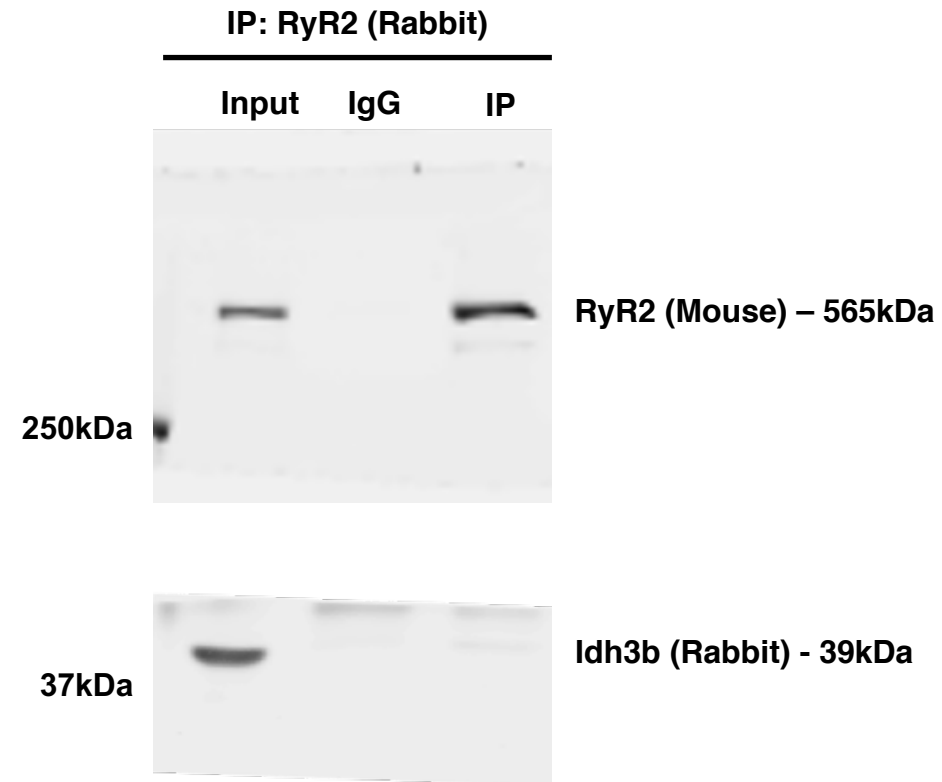

**Fig 4A (Black box demonstrates lane showed in figure)**

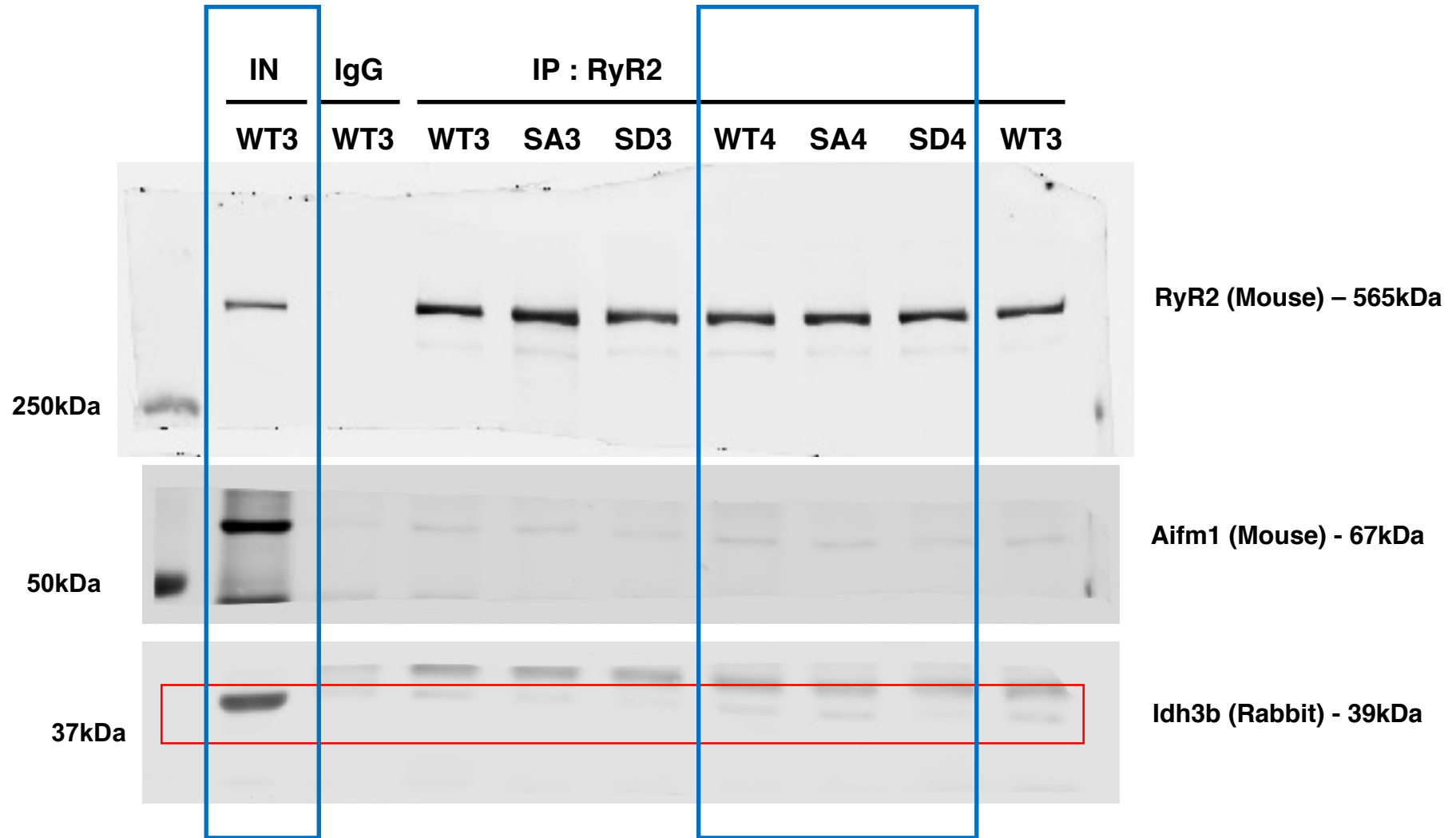

Supplement: Supplementary file 1 [file proteomes-09-00027-s001.zip › proteomes-1232014-original western.pdf]
